# Etanercept-Induced Cutaneous Necrotizing Vasculitis in a Patient With Rheumatoid Arthritis: A Case Report and Literature Review

**DOI:** 10.7759/cureus.116548

**Published:** 2026-09-19

**Authors:** Chaimae Nouri, Latifa Tahiri, Hanan Rkain, Fatine Kronbi, Fadoua Allali

**Affiliations:** 1 Department of Rheumatology B, Ayachi Hospital, Faculty of Medicine and Pharmacy of Rabat, Mohammed V University, Rabat, MAR; 2 Laboratory of Physiology, Mohammed V University, Faculty of Medicine and Pharmacy of Rabat, Rabat, MAR

**Keywords:** cutaneous vasculitis, drug-induced vasculitis, etanercept, necrotizing angiitis, rheumatoid arthritis

## Abstract

Tumor necrosis factor-alpha (TNF-α) inhibitors are effective therapies for rheumatoid arthritis (RA) but may rarely induce paradoxical autoimmune complications, including vasculitis. We report the case of a 53-year-old woman with seropositive RA and secondary Sjögren’s syndrome who developed bilateral dependent petechial and purpuric lesions of the lower extremities after approximately 33 months of etanercept therapy at a dose of 25 mg twice weekly. At symptom onset, her RA was well controlled, with no clinical or ultrasonographic synovitis and a Disease Activity Score in 28 joints of 2.1. Skin biopsy demonstrated fibrinoid necrosis of dermal vessels, consistent with necrotizing angiitis. No gastrointestinal, renal, neurological, pulmonary, or ear, nose, and throat involvement was identified. The etiologic workup showed a normal complete blood count, preserved renal function, normal urinalysis and complement levels, negative anti-myeloperoxidase, anti-proteinase 3, anti-double-stranded DNA antibodies and cryoglobulins, and negative hepatitis B, hepatitis C, and human immunodeficiency virus serologies. Serum protein electrophoresis showed no monoclonal gammopathy. Antinuclear antibodies were weakly positive at a titer of 1:80 without clear pathological significance. Etanercept was permanently discontinued, and the active lesions resolved completely within approximately 20 days, leaving only residual hyperpigmented and cicatricial changes. No recurrence was documented during follow-up. Rechallenge was not performed, and tocilizumab was subsequently selected for RA control. The compatible latency, histological confirmation, positive dechallenge, sustained absence of recurrence, and lack of a more plausible alternative cause strongly supported the diagnosis of cutaneous necrotizing vasculitis probably induced by etanercept. This case highlights that anti-TNF-α-associated vasculitis may occur after prolonged treatment and requires systematic assessment for systemic involvement and competing autoimmune, infectious, and hematologic causes.

## Introduction

Tumor necrosis factor-alpha (TNF-α) inhibitors, including etanercept, are established biologic disease-modifying antirheumatic drugs used in the management of rheumatoid arthritis, particularly in patients with an inadequate response or intolerance to conventional synthetic therapies [1]. These agents have substantially improved disease control and limited progressive structural damage.

Despite their therapeutic efficacy, TNF-α inhibitors may paradoxically induce immune-mediated adverse events, including lupus-like syndromes and vasculitis [2]. Cutaneous small-vessel vasculitis is the most frequently reported vasculitic manifestation and commonly presents as purpura of the lower extremities, although extracutaneous involvement may also occur [2,3]. Establishing drug causality can be challenging in patients with rheumatoid arthritis because vasculitis may also result from the underlying autoimmune disease or another secondary cause.

We report a case of cutaneous necrotizing vasculitis probably induced by etanercept in a patient with seropositive rheumatoid arthritis. This case highlights the importance of excluding systemic involvement and alternative etiologies and of discontinuing the suspected biologic agent.

## Case presentation

A 53-year-old woman with seropositive rheumatoid arthritis (RA), diagnosed in 2018, and secondary Sjögren’s syndrome presented in March 2022 with a new-onset purpuric eruption involving both lower extremities. Her medical history also included hypertension treated with amlodipine. She had no known history of cutaneous or systemic vasculitis before etanercept initiation.

Her RA was initially treated with methotrexate at a dose of 25 mg weekly, which was discontinued because of severe gastrointestinal intolerance, mainly recurrent vomiting. Leflunomide was subsequently initiated but was discontinued because of worsening hypertension. She had also received low-dose corticosteroid therapy. Because of intolerance or inadequate response to conventional synthetic disease-modifying antirheumatic drugs, etanercept was initiated in June 2019 at a dose of 25 mg twice weekly. Treatment provided satisfactory control of inflammatory arthritis for approximately three years. At the time of the cutaneous event, RA was clinically well controlled, with no tender or swollen joints. Serological testing showed a rheumatoid factor level of 80 IU/mL and an anti-cyclic citrullinated peptide (anti-CCP) antibody level of 92 U/mL, confirming seropositive rheumatoid arthritis. The erythrocyte sedimentation rate was 25 mm/h (reference range: 25-30 mm/h at the first hour), the C-reactive protein level was 2.42 mg/L (reference value: <6 mg/L), and the Disease Activity Score in 28 joints was 2.1 [4].

In March 2022, the patient developed bilateral dependent petechial and purpuric lesions over the lower legs, progressively extending to both knees. No gastrointestinal, renal, neurological, pulmonary, or ear, nose, and throat manifestations suggestive of systemic vasculitis were identified. No other recently introduced medication temporally associated with the eruption was documented (Figure 1).

**Figure 1:**
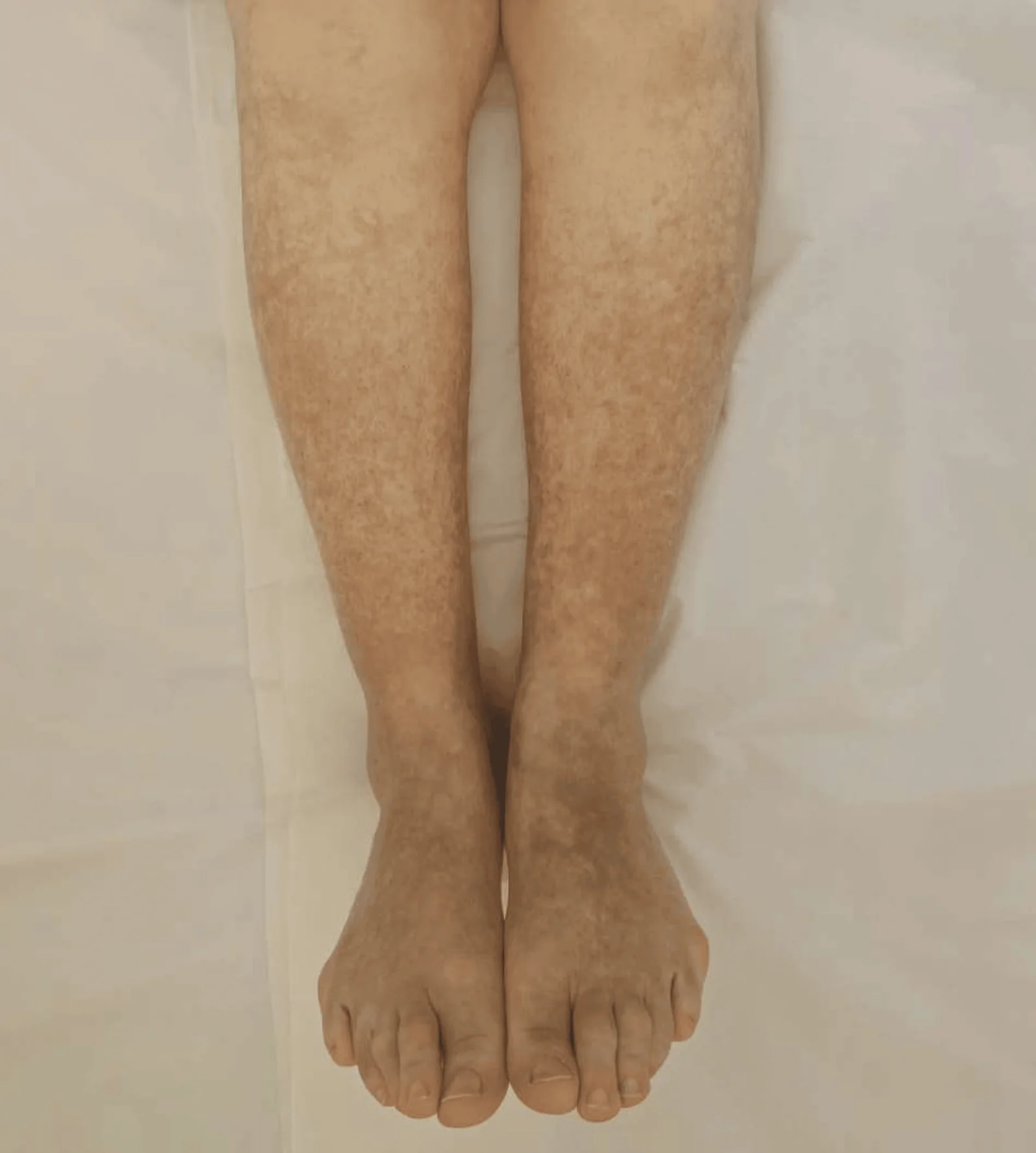
Residual Cutaneous Lesions Following Resolution of Etanercept-Associated Purpura Clinical photograph showing bilateral residual hyperpigmented and cicatricial lesions involving the lower extremities after complete resolution of the active purpuric eruption. Written informed consent was obtained from the patient for publication of the image.

According to the original pathology report, a skin biopsy performed in March 2022 showed a mild mononuclear inflammatory infiltrate composed predominantly of mature lymphocytes and histiocytes, focal thickening of the dermal capillary network, and definite areas of fibrinoid necrosis. The pathological conclusion was necrotizing angiitis. Direct immunofluorescence was not performed at the time of biopsy and could not subsequently be completed.

Because the eruption developed after nearly three years of well-tolerated etanercept therapy, a drug-related adverse event was not initially considered the most likely explanation, and etanercept was continued during the early diagnostic assessment. Suspicion of etanercept-induced vasculitis increased after the skin biopsy demonstrated necrotizing angiitis, while the etiological workup did not identify a more plausible autoimmune, infectious, cryoglobulinemic, or hematologic cause and the underlying RA remained clinically well controlled. In this context, etanercept was considered the most likely trigger and was permanently discontinued in June 2022 (Table 1).

**Table 1:** Clinical and Therapeutic Timeline RA, rheumatoid arthritis; DAS28, Disease Activity Score in 28 joints.

Date/period	RA status and treatment	Cutaneous event and investigations	Management and outcome
2018	Seropositive RA diagnosed	No previous cutaneous or systemic vasculitis	Conventional treatment initiated
Before June 2019	Methotrexate discontinued because of severe gastrointestinal intolerance; leflunomide subsequently discontinued because of worsening hypertension	No vasculitic manifestations documented	Alternative biologic therapy considered
June 2019	Etanercept initiated at 25 mg twice weekly	No initial cutaneous adverse event	Satisfactory control of inflammatory arthritis
March 2022	RA clinically controlled; no clinical or ultrasonographic synovitis; DAS28: 2.1	Bilateral dependent petechial and purpuric lesions extending to the knees; biopsy showed fibrinoid necrosis consistent with necrotizing angiitis	Dermatological evaluation and etiological assessment initiated
June 2022	Etanercept permanently discontinued after the initial diagnostic assessment	Clinical and histological findings supported a probable drug-related vasculitis	Etanercept withdrawn; no rechallenge performed
Approximately 20 days after withdrawal	-	Complete resolution of active purpuric lesions	Residual hyperpigmented and cicatricial lesions persisted
Subsequent follow-up	No recurrence after etanercept withdrawal	Dermatological reassessment showed inactive residual lesions	Permanent avoidance of etanercept recommended
Later therapeutic course	Ongoing need for RA disease control	No recurrent purpura documented	Tocilizumab selected as an alternative biologic therapy; RA remained clinically well controlled during follow-up.

An etiological workup was performed to investigate alternative causes of cutaneous vasculitis. The complete blood count and platelet count were normal, as were serum C3 and C4 complement levels. Renal function was preserved, and urinalysis showed neither hematuria nor proteinuria. Serological testing for hepatitis B virus, hepatitis C virus, and human immunodeficiency virus was negative. Cryoglobulin testing remained negative after four weeks of observation.

Anti-myeloperoxidase and anti-proteinase 3 antibodies were negative. Antinuclear antibodies were weakly positive at a titer of 1:80 with a speckled pattern, whereas anti-double-stranded DNA antibodies were negative. Serum protein electrophoresis showed an inflammatory pattern, with increased alpha-1 and alpha-2 globulin fractions, but no monoclonal gammopathy (Table 2).

**Table 2:** Etiological Workup for Cutaneous Vasculitis ANCA, Antineutrophil cytoplasmic antibodies; MPO, myeloperoxidase; PR3, proteinase 3; ENT, ear, nose, and throat; HBV, hepatitis B virus; HCV, hepatitis C virus; HIV, human immunodeficiency virus.

Diagnostic domain	Investigation	Result	Interpretation
Hematologic	Hemoglobin	13.0 g/dl	No cytopenia or leukocytosis, suggesting an alternative systemic process
	White blood cells (WBC)	4,960/mm³	
Hematologic	Platelet count	223,000/mm^3^	Thrombocytopenic purpura unlikely
Renal	Serum creatinine	7.2 mg/L	Preserved renal function
Renal	Urinalysis	No hematuria	No evidence of glomerular involvement
Renal	Proteinuria assessment	Negative	No documented renal involvement
Complement	C3	Within normal limits, 1.1 g/L	No complement consumption
Complement	C4	Within normal limits, 0.3 g/L	Cryoglobulinemic vasculitis less likely
ANCA-associated vasculitis	Anti-myeloperoxidase antibodies	Negative, 0.5 U/mL	No serological support for MPO-ANCA-associated vasculitis
ANCA-associated vasculitis	Anti-proteinase 3 antibodies	Negative, 1.4 U/mL	No serological support for PR3-ANCA-associated vasculitis
Connective tissue disease	Antinuclear antibodies	Weakly positive, speckled pattern, 1:80	Low-titer result without clear pathological significance
Connective tissue disease	Anti-double-stranded DNA antibodies	Negative, 12.9 IU/mL	No serological support for active systemic lupus erythematosus
Cryoglobulinemia	Serum cryoglobulins	Negative after four weeks	Cryoglobulinemic vasculitis less likely
Monoclonal gammopathy	Serum protein electrophoresis	Inflammatory pattern; no monoclonal gammopathy	No evidence of an underlying monoclonal gammopathy
Infection	Hepatitis B virus serology	Negative	HBV-related vasculitis unlikely
Infection	Hepatitis C virus serology	Negative	HCV-associated cryoglobulinemic vasculitis unlikely
Infection	Human immunodeficiency virus serology	Negative	HIV-associated vasculitis unlikely
Clinical systemic assessment	Gastrointestinal, neurological, pulmonary, ENT, and renal manifestations	None documented	Findings supported a skin-limited vasculitic episode

The temporal relationship between etanercept exposure and the development of purpura, the histological evidence of necrotizing angiitis, the complete resolution of active lesions after drug withdrawal, the absence of recurrence after withdrawal and without re-exposure, and the lack of an identified alternative systemic cause supported the diagnosis of cutaneous necrotizing vasculitis probably induced by etanercept.

Because re-exposure to etanercept was considered inappropriate, tocilizumab, a biologic agent with a different mechanism of action, was subsequently selected for RA management. Rheumatoid arthritis remained clinically well controlled during follow-up.

## Discussion

TNF-α inhibitors have profoundly transformed the management of rheumatoid arthritis; however, their use may paradoxically be associated with de novo autoimmune manifestations, including lupus-like syndromes, autoantibody induction, and vasculitis [1,2]. Although anti-TNFα-associated vasculitis remains uncommon, its relationship with this therapeutic class is now well documented. In the retrospective series by Sokumbi et al., eight cases of vasculitis associated with TNFα inhibitors were identified, including two related to etanercept. The skin was the most frequently involved organ, affecting five of the eight patients, and palpable purpura was the predominant cutaneous manifestation. All cases were histologically confirmed. Systemic involvement, mainly peripheral neurological disease and, less frequently, renal involvement, was also reported, emphasizing the need to assess for extracutaneous disease in any patient who develops purpura while receiving anti-TNF-α therapy [3]. The French nationwide survey by Saint Marcoux and De Bandt identified 39 cases, including 21 associated with etanercept, whereas an analysis of the United States pharmacovigilance database reported 35 cases of leukocytoclastic vasculitis, 20 of which were linked to etanercept [5,6]. More recently, an analysis of 164 cases from the French national pharmacovigilance database confirmed the predominance of cutaneous manifestations, which occurred in 88% of cases and most commonly presented as purpura [7]. Collectively, these findings support a paradoxical adverse effect associated with the anti-TNF-α class, with etanercept being repeatedly implicated.

The presentation of our patient was consistent with the phenotype most frequently described in the literature. The lesions were bilateral, dependent, and confined to the lower extremities, extending to the knees, without documented visceral involvement. Palpable or petechial purpura, typically symmetrical and predominantly affecting the lower limbs, is the characteristic clinical presentation of cutaneous small-vessel vasculitis [8,9]. Nevertheless, in the recent French pharmacovigilance analysis, renal involvement was reported in 14% of cases, while gastrointestinal and neurological manifestations were each observed in 8% [7]. These findings underscore the importance of systematically screening for systemic involvement even when the initial presentation appears to be limited to the skin.

Compatibility of the time to onset

Etanercept was initiated in June 2019, and the first cutaneous lesions appeared in March 2022, after approximately 33 months of exposure. This relatively long latency does not argue against a drug-related mechanism. In the historical French series, the mean duration of exposure to TNF-α inhibitors before the onset of vasculitis was 9.6 months, although substantial variability was reported [5]. In the French pharmacovigilance analysis published in 2026, which included 164 cases, the median time to onset of vasculitis was 390 days, with an interquartile range of 120 to 1,095 days [7]. Therefore, the 33-month latency observed in our patient remains consistent with previously published data. This case highlights that an initially favorable safety profile, even over several years, does not exclude the delayed occurrence of an immune-mediated complication.

Several published reports have specifically described an association between etanercept and vasculitis. As early as 2000, Galaria et al. reported a case of leukocytoclastic vasculitis occurring during etanercept therapy, with complete resolution after treatment withdrawal [10]. More severe systemic presentations, including ANCA-associated necrotizing glomerulonephritis and immune complex-mediated glomerulonephritis, have also been described during anti-TNF-α therapy [11,12]. These reports illustrate that the clinical spectrum may range from isolated cutaneous disease to multiorgan vasculitis, supporting the systematic assessment of renal, neurological, gastrointestinal, and pulmonary involvement, even when the initial lesions appear confined to the skin.

Histopathological findings and limitations

The diagnosis of cutaneous vasculitis relies on the integration of clinical, laboratory, and histopathological findings rather than on any single feature [8,9]. In our patient, skin biopsy demonstrated fibrinoid necrosis involving dermal vessels, leading to the pathological conclusion of “necrotizing angiitis.”The pathology report described involvement of the dermal capillary network but did not explicitly specify the caliber of the affected vessels. Therefore, a definitive histopathological classification as small-vessel versus medium-vessel vasculitis could not be made from the available report. Fibrinoid necrosis provides an objective evidence of inflammatory and destructive injury to the vessel wall and, in conjunction with the purpuric presentation, supports the diagnosis of cutaneous necrotizing vasculitis.

However, the available histopathology report did not explicitly describe a neutrophilic infiltrate, leukocytoclasia, or erythrocyte extravasation. It was therefore considered more accurate to retain the descriptive diagnosis of cutaneous necrotizing vasculitis rather than classify the lesion as leukocytoclastic vasculitis, a pattern that was not specifically reported by the pathologist. The Chapel Hill Consensus Conference nomenclature and its dermatologic addendum emphasize the distinction between the observed histopathological pattern, the presumed etiology, and whether the vasculitis is confined to the skin or represents part of a systemic disorder [13,14].

Direct immunofluorescence was not performed at the time of the initial biopsy and could not be completed retrospectively. Its absence does not invalidate the histological diagnosis of necrotizing vasculitis but limits its immunopathological classification. When immune complex-mediated cutaneous vasculitis is suspected, direct immunofluorescence should ideally be performed on an early lesion, preferably a recent partially blanchable macule, to assess vascular deposits of immunoglobulins and complement components [9].

Assessment of etanercept causality

Drug causality in cutaneous vasculitis cannot be established by a single diagnostic test. Instead, it relies on the convergence of several elements, including a compatible temporal relationship, clinical and histopathological consistency, improvement following drug withdrawal, previously reported associations, and the reasonable exclusion of competing causes [15].

Several findings supported etanercept as the most likely trigger in our patient. She had no known history of vasculitis before starting etanercept, and the purpuric eruption developed during treatment. Skin biopsy provided objective evidence of necrotizing angiitis. Following permanent discontinuation of etanercept, the active lesions completely resolved within approximately 20 days, leaving only residual hyperpigmented and cicatricial changes. No recurrence was documented during follow-up in the absence of re-exposure, and the etiological workup did not identify a more plausible alternative cause.

The positive dechallenge, defined as improvement after withdrawal of the suspected drug, represents the strongest chronological argument in this case. In the pharmacovigilance analysis by Mohan et al., 22 of 35 patients (62.8%) experienced complete or marked improvement of cutaneous lesions after discontinuation of the TNF-α inhibitor. Six patients developed recurrent vasculitis after treatment was restarted, providing additional evidence supporting a causal association [6]. In our patient, deliberate rechallenge was neither necessary nor ethically justified because the lesions had resolved after withdrawal and re-exposure could have resulted in recurrence or a potentially more severe reaction.

The status of the underlying rheumatoid arthritis further strengthens this interpretation. At the time of the cutaneous event, the patient had no clinical or ultrasonographic synovitis, with tender and swollen joint counts of zero, a C-reactive protein level of 2.42 mg/L, and a Disease Activity Score in 28 joints of 2.1. This dissociation between well-controlled articular disease and the development of a new vasculitic eruption makes uncontrolled rheumatoid inflammation a less convincing explanation, although it does not entirely exclude rheumatoid vasculitis. Previous series evaluating anti-TNF-α-associated vasculitis similarly considered quiescence of the underlying disease and the absence of a more likely alternative cause as important attribution criteria [3].

Taken together, the compatible temporal relationship, histopathological confirmation, rapid and sustained resolution after etanercept withdrawal, absence of recurrence without re-exposure, controlled rheumatoid arthritis, and exclusion of the principal competing etiologies strongly support the diagnosis of cutaneous necrotizing vasculitis probably induced by etanercept.

Exclusion of the main competing etiologies

Rheumatoid vasculitis was an important differential diagnosis because of the seropositive nature of the patient’s rheumatoid arthritis. However, it most commonly develops in patients with longstanding, severe disease and is frequently associated with other extra-articular manifestations. In the case-control study by Makol et al., the median duration of RA before the onset of vasculitis was 10.8 years, and most patients had seropositive disease with elevated inflammatory markers [16]. In our patient, RA had been present for approximately four years and was well controlled at the time of the eruption, with no clinical or ultrasonographic synovitis and no neurological, digital, ulcerative, or visceral features suggestive of rheumatoid vasculitis. Nevertheless, control of articular disease alone does not exclude this diagnosis. In our patient, the relatively short duration of RA, low disease activity, rapid resolution of purpura after etanercept withdrawal, and absence of recurrence made rheumatoid vasculitis less likely.

The presence of secondary Sjögren’s syndrome also required the exclusion of cryoglobulinemic vasculitis. In studies of Sjögren’s syndrome, cryoglobulinemic vasculitis commonly presents with purpura and may be associated with low C4 levels, peripheral neuropathy, leukopenia, a monoclonal component, and an increased risk of lymphoproliferative complications [17,18]. In our patient, cryoglobulin testing remained negative after four weeks, C3 and C4 levels were normal, the complete blood count showed neither leukopenia nor thrombocytopenia, and serum protein electrophoresis demonstrated no monoclonal gammopathy. The absence of peripheral neuropathy, hematuria, proteinuria, or impaired renal function further argued against this diagnosis. Continued surveillance of the mediastinal lymphadenopathy nevertheless remained warranted in the context of Sjögren’s syndrome, independently of the etiology of the cutaneous episode [17,18].

ANCA-associated vasculitis was also considered unlikely because anti-MPO and anti-PR3 antibodies were negative, renal function was preserved, urinalysis showed neither hematuria nor proteinuria, and no ear, nose, and throat, pulmonary, or neurological manifestations were present [8,9]. Antinuclear antibodies were weakly positive with a speckled pattern at a titer of 1:80, whereas anti-double-stranded DNA antibodies were negative and complement levels were normal. TNF-α inhibitors may induce antinuclear antibodies and, less frequently, anti-DNA antibodies without such serological autoimmunity necessarily indicating a clinical lupus-like syndrome [2,19]. Therefore, this isolated low-titer positivity, in the absence of systemic manifestations or complement consumption, did not support etanercept-induced lupus. Finally, serological testing for hepatitis B virus, hepatitis C virus, and human immunodeficiency virus was negative, and no concurrent infectious focus was identified, making infection-related immune complex vasculitis unlikely [8,9].

Overall, the workup did not identify a more convincing autoimmune, cryoglobulinemic, infectious, or hematologic cause. This reasonable exclusion of the main competing etiologies, together with compatible histopathology and a positive dechallenge, further supported the probable attribution of the vasculitis to etanercept, although absolute causality could not be established.

Potential pathophysiological mechanisms

The mechanisms underlying paradoxical vasculitis during TNF-α inhibitor therapy remain incompletely understood. One proposed mechanism involves the development of antibodies against the biological agent, followed by the formation and vascular deposition of immune complexes, with subsequent complement activation and small-vessel inflammation. Other hypotheses include disruption of the cytokine balance with enhanced type I interferon signaling, induction of autoantibodies, increased neutrophil apoptosis, and the formation or exposure of neoantigens [3,15,19]. These mechanisms remain hypothetical and are likely heterogeneous, depending on the TNF-α inhibitor used, the underlying disease, and the clinical and histopathological phenotype of vasculitis.

Management and clinical outcome

Withdrawal of the suspected medication is the cornerstone of the management of drug-induced vasculitis [6,7,15]. Skin-limited forms are often self-limiting and may resolve within three to four weeks without systemic therapy. Short-term systemic glucocorticoids may be considered for painful, ulcerative, extensive, or otherwise severe cutaneous disease, whereas organ-threatening or systemic involvement may require additional immunosuppressive treatment tailored to the affected organs and disease severity [15,20].

In our patient, etanercept was permanently discontinued, and the active cutaneous lesions resolved completely within approximately 20 days, leaving only residual hyperpigmented and cicatricial changes. No vasculitis-specific systemic treatment was documented. The rapid improvement following drug withdrawal, together with the sustained absence of recurrence, was consistent with a skin-limited drug-induced vasculitis and further strengthened the suspected causal relationship.

A subsequent dermatological assessment confirmed the absence of active lesions and recommended against re-exposure to etanercept. Although the available evidence does not establish that all TNF-α inhibitors are invariably contraindicated after an etanercept-associated vasculitic event, recurrence after reintroduction has been reported, and recent pharmacovigilance data support discontinuation of the suspected agent whenever possible [6,7].

Tocilizumab, which targets the interleukin-6 receptor and therefore has a different mechanism of action, was subsequently selected for continued control of rheumatoid arthritis. In the absence of robust comparative evidence regarding the safest subsequent biological therapy, switching to a different therapeutic class represented a cautious and individualized strategy aimed at avoiding re-exposure to the suspected trigger.

Strengths and limitations

The main limitation of this case was the absence of direct immunofluorescence, which prevented precise immunopathological classification of the vasculitis. The histopathology report was also relatively limited and did not provide a detailed characterization of the inflammatory infiltrate. The original histological slides and micrographs were not available for retrospective review. Consequently, a histopathological figure could not be provided. Rechallenge was not performed, but it was neither necessary nor ethically appropriate given the clinical improvement after etanercept withdrawal and the potential risk of recurrence.

The main strengths were histological documentation of necrotizing angiitis, a compatible temporal relationship, well-controlled rheumatoid arthritis at the time of the event, rapid resolution after etanercept withdrawal, absence of recurrence, and exclusion of the major competing diagnoses. Although none of these findings alone proves causality, their convergence strongly supports the diagnosis of cutaneous necrotizing vasculitis probably induced by etanercept.

This case emphasizes that drug-induced vasculitis should be considered in any patient developing purpura during anti-TNF-α therapy, even after several years of good tolerance. Evaluation should confirm vascular involvement, screen for systemic disease, exclude alternative infectious, autoimmune, and hematologic causes, and assess the clinical response after withdrawal of the suspected drug.

## Conclusions

Etanercept-associated vasculitis should be considered in patients who develop new purpuric lesions during anti-TNF-α therapy, even after several years of apparently good tolerance. In this case, the compatible chronology, histological evidence of necrotizing angiitis, absence of a more convincing alternative cause, and complete resolution after etanercept withdrawal strongly supported the diagnosis of cutaneous necrotizing vasculitis probably induced by etanercept. A systematic evaluation for systemic involvement and competing autoimmune, infectious, and hematologic causes remains essential. Permanent discontinuation of the suspected agent and switching to a biologic with a different mechanism of action may represent the safest therapeutic strategy.
